# Health Information Technology Use Among Persons With Self-reported Atherosclerotic Cardiovascular Disease: Analysis of the 2011-2018 National Health Interview Survey

**DOI:** 10.2196/23765

**Published:** 2021-08-13

**Authors:** Uchenna Nwokeji, Erin M Spaulding, Rongzi Shan, Ruth-Alma Turkson-Ocran, Diana Baptiste, Binu Koirala, Timothy B Plante, Seth S Martin, Yvonne Commodore-Mensah

**Affiliations:** 1 Department of Pharmacology and Cellular Biology University of Pittsburgh School of Medicine Pittsburgh, PA United States; 2 Johns Hopkins University School of Nursing Baltimore, MD United States; 3 The Welch Center for Prevention Epidemiology and Clinical Research Johns Hopkins University Bloomberg School of Public Health Baltimore, MD United States; 4 Center for Mobile Technologies to Achieve Equity in Cardiovascular Health American Heart Association Strategically Focused Research Network Health Technology and Innovation Center Baltimore, MD United States; 5 Department of Medicine David Geffen School of Medicine Los Angeles, CA United States; 6 Division of General Internal Medicine Johns Hopkins University School of Medicine Baltimore, MD United States; 7 Department of Medicine Larner College of Medicine at the University of Vermont Burlington, VT United States; 8 Ciccarone Center for the Prevention of Cardiovascular Disease Division of Cardiology, Department of Medicine Johns Hopkins University School of Medicine Baltimore, MD United States; 9 Department of Epidemiology Johns Hopkins Bloomberg School of Public Health Baltimore, MD United States

**Keywords:** health information technology, cardiovascular disease, digital health, eHealth, mobile phone

## Abstract

**Background:**

Atherosclerotic cardiovascular disease (ASCVD) is the leading cause of morbidity and mortality in the United States. Health information technologies (HITs) have recently emerged as a viable intervention to mitigate the burden of ASCVD. Approximately 60% of US adults report searching the internet for health information; however, previous research has not examined the prevalence of general technology or HIT use among adults with and without ASCVD. In addition, social determinants in HIT use among adults with ASCVD are not well understood.

**Objective:**

The aim of this study was to evaluate the prevalence and social determinants of HIT use among US adults with versus without self-reported ASCVD.

**Methods:**

We pooled cross-sectional data from the 2011-2018 National Health Interview Survey (NHIS) to examine the general technology and HIT use among adults aged ≥18 years with and without self-reported ASCVD (coronary heart disease, stroke, or both). General technology use was defined as mobile phone ownership, internet use, and computer use. HIT use was defined as looking up health information on the internet, filling a web-based prescription, scheduling a medical appointment on the internet, communicating with a health care provider by email, or using web-based group chats to learn about health topics. We evaluated sociodemographic differences in HIT use among respondents by using Poisson regression. Analyses were weighted according to NHIS standards.

**Results:**

A total sample of 256,117 individuals were included, of which 2194 (0.9%) reported prior ASCVD. Among adults with prior ASCVD, the mean age was 70.6 (SD 11.5) years, and 47.4% (1048/2194) of the adults were females. General technology use differed between participants with and without prior ASCVD, with 36.0% (614/1826) and 76.2% (157,642/213,816) indicating internet usage and 24.6% (374/1575) and 60.7% (107,742/184,557) indicating using a computer every day, respectively. Similarly, adults with ASCVD were less likely to use HIT than those without ASCVD (515/2194, 25.1% vs 123,966/253,923, 51.0%; *P*<.001). Among adults with prior ASCVD, social determinants that were associated with HIT use included younger age, higher education, higher income, being employed, and being married.

**Conclusions:**

HIT use was low among adults with a history of ASCVD, which may represent a barrier to delivering care via emerging HIT. Given the associations with social determinants such as income, education, and employment, targeted strategies and policies are needed to eliminate barriers to impact HIT usage.

## Introduction

Atherosclerotic cardiovascular disease (ASCVD) is the leading cause of morbidity and mortality in the United States, accounting for more lives lost per year than those lost to cancer and chronic lung disease combined [1]. In 2016, over 360,000 people died from coronary heart disease, which is the most prevalent form of heart disease. Additionally, 1 in every 19 deaths in the United States, on average, was due to stroke. From 2014 to 2015, the economic burden associated with ASCVD was estimated to result in US $351.2 billion in direct and indirect annual costs [1]. Adults with prior ASCVD events are at high risk for recurrence and require contemporary secondary prevention strategies, including behavioral and medical interventions [2]. ASCVD outcomes are also disparate among groups of persons with different social determinants, including persons with lower income, lower education, and racial minority status [3]. Novel technologies may play a role in addressing the barriers contributing to disparities in ASCVD outcomes.

Health information technologies (HITs) have recently emerged as a viable intervention to mitigate the burden of ASCVD [4,5]. HITs, which encompass patient portals, mobile phone interventions, electronic health records, and telemedicine services, are increasingly being used to improve communication between patients and clinicians and to facilitate chronic disease management [6,7]. Approximately 60% of US adults report searching the internet for health information [8]. Around late March/early April 2020, the implementation of telemedicine services to meet patient demand drastically increased in response to the COVID-19 pandemic, which has reached 92,262,621 cases in 223 countries as of January 16, 2021 [9-11]. Historically, however, there has been a “digital divide” in which underserved populations lack access to computers and the internet, thereby serving as a significant barrier to care management utilizing HIT [12]. In recent years, mobile phones have helped in bridging this divide. In terms of options for online access, approximately 25% of Hispanics and 23% of Non-Hispanic Blacks are “smartphone only” internet users in place of traditional home broadband services compared to only 12% of Whites [13]. Non-Hispanic Blacks and Hispanics are also more likely than Non-Hispanic Whites to seek health information via their smartphones [8].

Previous studies on the sociodemographic characteristics of general US adult population using HIT have found that these adults tend to be Whites, women, young, and have a higher income and education level [14-16]. In terms of HIT use among the CVD population, 73% of US adults with or at risk for CVD owned a smartphone and 48% had a health app [17]. Adults with or at risk for CVD were also more likely to share health information from a smartphone/wearable device with a clinician [17]. Few studies utilizing nationally representative databases have assessed sociodemographic differences in HIT use among adults with self-reported ASCVD. To better understand the differences in HIT use among adults with ASCVD, we analyzed the National Health Interview Survey (NHIS). Specifically, we sought to (1) evaluate general technology and HIT use by prior ASCVD status, (2) describe changes in HIT use over time, and (3) describe social determinants of HIT use among US adults with ASCVD. We hypothesized that among adults with prior ASCVD, (1) general technology and HIT use would be lower, (2) HIT use would increase over time, and (3) social determinants of health that are indicative of more vulnerable status would be associated with lower HIT use.

## Methods

### Study Population

Analyses were performed with cross-sectional data from the NHIS, a civilian noninstitutionalized population survey of US adults aged ≥18 years, which was administered by the National Center for Health Statistics (NCHS). From 2006 to 2015, NHIS employed a multistage stratified cluster probability design that oversampled Black, Hispanic, and Asian people; however, oversampling was stopped in 2016 to adjust for the changes in the distribution of the US population since 2006. NHIS also reaches 35,000 households with about 87,500 persons annually [18]. For the sample adult questionnaire, one randomly selected adult per family is interviewed in person by a trained NCHS staff member who records the participant’s self-reported information on health care access and utilization, health status, behavior, and other sociodemographic data [18]. A full description of the NHIS methodologies can be found elsewhere [19]. The data for the years 2011-2018 were pooled using NCHS guidelines to improve the accuracy of the estimates [18].

### Participants

Respondents included in the analysis were US adults aged ≥18 years who answered “yes” or “no” to “Have you ever been told by a doctor or other health professional that you had coronary heart disease?” We also included US adults who answered “yes” or “no” to “Have you ever been told by a doctor or other health professional that you had a stroke?” We defined ASCVD as self-reported coronary heart disease, stroke, or both. Of the 257,653 participants in NHIS, we excluded participants who did not provide data on education (n=1115), employment (n=142), and health status (n=279). The analytical sample included 256,117 participants.

### Outcome Measurements

#### General Technology Use

Mobile phone ownership, internet use, and computer use were compared between US adults with and without ASCVD to assess the prevalence of general technology use. Mobile phone ownership was derived from persons who responded “≥1” to the question: “How many working cell phones do you or people in your family have?” Internet use was derived from persons who answered “yes” to the question: “Do you use the internet?” Computer use was derived from persons who answered, “Never or almost never,” “Some days,” “Most days,” or “Every day” to the question: “How often do you use a computer?”

#### HIT Use

HIT use, the primary outcome, was defined as responding “yes” from the responses “yes,” “no,” “refused,” “not ascertained,” or “don’t know” to a question regarding computer use in the prior 12 months to do one of the following: looking up health information on the internet, filling a web-based prescription, scheduling a medical appointment on the internet, communicating with health care providers through email, or using web-based group chats to learn about health topics. 

### Covariates

Covariates included self-reported age, sex, race/ethnicity, education level, employment status, health insurance status, health status, marital status, and income. Education level was recorded as ≤high school, some college, or ≥bachelor's degree. Health insurance status responses were categorized as covered and not covered. Health status was defined as reporting feeling better, worse, or about the same compared to last year. Income was measured by poverty income ratio, which is a variable calculated by the NCHS using the midpoint family income divided by the poverty level in dollars, corresponding to the US Census Bureau of the same survey year.

### Statistical Analysis

Sample weights recommended by NCHS for the analytic years were used to adjust for the complex sample design [19]. We examined demographic characteristics by survey-weighted percentages among adults with and without a history of ASCVD. Weighted percentages were also calculated to measure the prevalence of general technology use and HIT use. Due to the complex sampling strategy, resulting in varying weights of individual observations, the percentage calculated by dividing the raw number of adults by the total n of the category of interest does not necessarily equal to the tabulated weighted percentage. Chi-square tests were used to examine differences in the HIT use categories, with *P* values <.05 deemed statistically significant. We examined the estimated prevalence of HIT use by using generalized linear models with a Poisson distribution and logarithmic link with linearized variance estimation. We also examined the adjusted predicted values and marginal effects of the primary outcome. The model was adjusted for age, sex, race/ethnicity, education level, employment status, health insurance status, health status, marital status, and income. Statistical analyses were performed with Stata version 16.0 SE (StataCorp LLC).

## Results

### Demographics of the Study Population

Of the 256,117 participants, 2194 (0.9%) reported prior ASCVD. Table 1 displays the prevalence of the demographic characteristics among the subpopulations of adults with and without a history of ASCVD. The mean age of the participants was 70.6 (SD 11.5) years, 47.4% (1048/2194) were females, and 55.1% (1270/2194) received no more than high school education. Compared to respondents without a history of ASCVD, adults with a history of ASCVD were also more often male, older, non-Hispanic White, had lower income, unemployed, less educated, uninsured, and at a worse overall health status from the previous year.

**Table 1:** Demographics of the adults with and without a history of atherosclerotic cardiovascular disease in the National Health Interview Survey.

Sociodemographic characteristics		Adults with ASCVD^a^ (n=2194), %^b^ (95% CI)	Adults without ASCVD (n=253,923), % (95% CI)
**Sex**			
	Male	52.6 (50.1-55.1)	45.8 (45.6-46.1)
	Female	47.4 (44.9-49.9)	54.2 (53.9-54.5)
**Age (years)**			
	70+	56.0 (53.6-58.4)	15.8 (15.5-16.1)
	60-69	27.5 (25.3-29.8)	15.9 (15.6-16.1)
	50-59	12.2 (10.7-13.9)	17.2 (16.9-17.4)
	40-49	3.1 (2.4-4.1)	15.7 (15.5-15.9)
	30-39	1.1 (0.6-1.8)	16.6 (16.4-16.8)
	18-29	0.1 (0.05-0.3)	18.9 (18.5-19.4)
**Race/ethnicity**			
	Non-Hispanic White	70.7 (68.3-72.9)	68.6 (67.9-69.2)
	Hispanic	9.4 (7.9-11.1)	13.0 (12.5-13.5)
	Non-Hispanic Black	15.9 (14.3-17.7)	12.5 (12.1-13.0)
	Non-Hispanic Asian	2.9 (2.2-3.7)	4.9 (4.7-5.1)
	Non-Hispanic multiple races and other races	1.1 (0.7-1.8)	1.0 (0.8-1.1)
**Poverty income ratio**			
	Below poverty level	22.5 (20.5-24.5)	15.2 (14.9-15.6)
	Between 100% and 200% of poverty level	30.9 (28.8-33.2)	19.0 (18.7-19.3)
	>200% above poverty level	46.6 (44.1-49.2)	65.8 (65.2-66.3)
**Employment status**			
	Not employed	91.5 (89.9-92.8)	41.5 (41.1-41.9)
	Employed	8.5 (7.2-10.1)	58.5 (58.1-58.9)
**Marital status**			
	Not married	62.9 (60.5-65.3)	56.1 (55.7-56.6)
	Married	37.1 (34.7-39.5)	43.9 (43.4-44.3)
**Health status**			
	Better	19.6 (17.7-21.6)	18.4 (18.2-18.6)
	Worse	26.3 (24.3-28.5)	8.5 (8.4-8.7)
	About the same	54.1 (51.6-56.6)	73.1 (72.9-73.3)
**Education level**			
	≤High school	55.1 (52.5-57.6)	37.2 (36.7-37.7)
	Some college	28.0 (25.9-30.3)	31.1 (30.8-31.5)
	≥Bachelor’s degree	16.9 (15.2-18.9)	31.7 (31.1-32.2)
**Insurance coverage**			
	Not covered	97.4 (96.4-98.1)	87.9 (87.7-88.2)
	Covered	2.6 (1.9-3.6)	12.1 (11.8-12.4)

^a^ASCVD: atherosclerotic cardiovascular disease.

^b^Survey-weighted percentages.

### Prevalence of Web Access and Electronic Device Ownership

**Figure 1:**
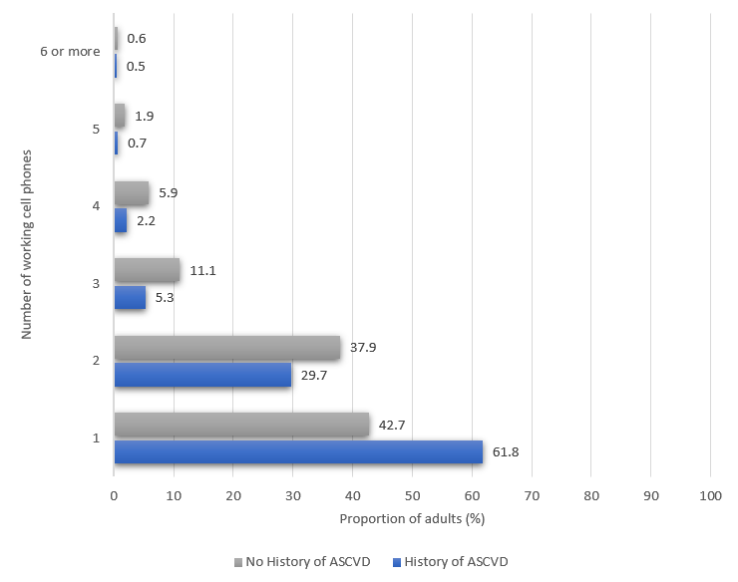
Mobile phone ownership among US adults with and without a history of atherosclerotic cardiovascular disease. ASCVD: atherosclerotic cardiovascular disease.

**Figure 2:**
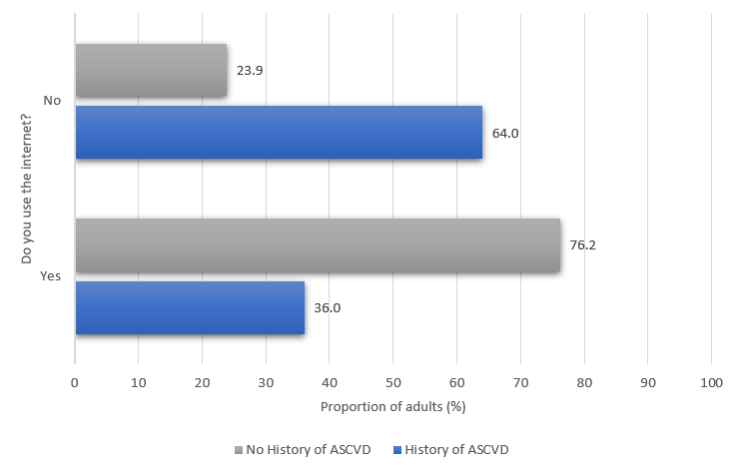
Internet use among US adults with and without a history of atherosclerotic cardiovascular disease. ASCVD: atherosclerotic cardiovascular disease.

**Figure 3:**
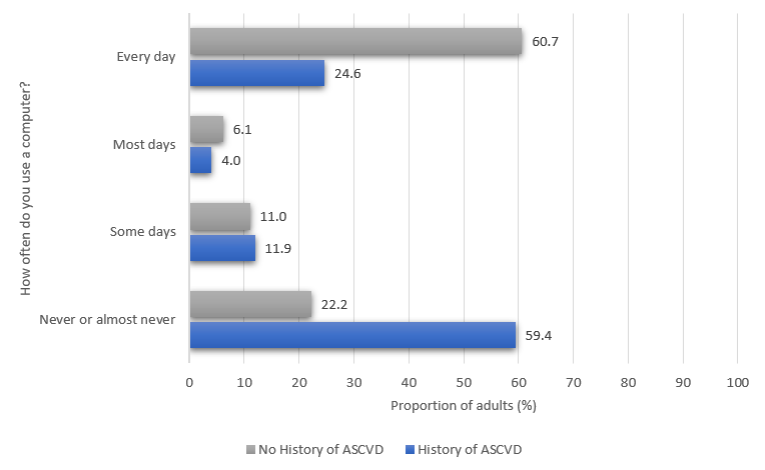
Frequency of computer use among US adults with and without a history of atherosclerotic cardiovascular disease. ASCVD: atherosclerotic cardiovascular disease.

Approximately 76.7% (1682/2194) of the US adults with ASCVD and 89.1% (226,357/253,923) of the US adults without ASCVD reported their household owning at least one mobile phone. Figure 1 compares mobile phone ownership among US adults with and without a history of ASCVD. Of 2194 US adults, 1826 (83.2%) adults with self-reported ASCVD reported on whether they used the internet. Of the total respondents, 36.0% (614/1826) of adults with a history of ASCVD indicated internet use. Approximately 84.2% (213,816/253,923) of the US adults without ASCVD reported on whether they used the internet. Of the total respondents, 76.2% (157,642/213,816) of adults without a history of ASCVD indicated internet use. Figure 2 compares internet use among US adults with and without a history of ASCVD. Approximately 71.8% (1575/2194) of the US adults with self-reported ASCVD reported on how often they use a computer. Of the total respondents, 24.6% (374/1575) of adults with a history of ASCVD indicated that they use a computer every day. Approximately 72.7% (184,557/253,923) of the US adults without ASCVD reported on how often they use a computer. Of the total respondents, 24.6% (374/1575) of adults with a history of ASCVD indicated that they use a computer every day. Approximately 72.6% (184,557/253,923) of the US adults without ASCVD reported on how often they use a computer. Of the total respondents, 60.7% (107,742/184,557) of adults without a history of ASCVD indicated that they use a computer every day. Figure 3 compares the frequency of computer use among US adults with and without a history of ASCVD.

### HIT Use Differences Between Those With and Those Without ASCVD

Of the respondents with a history of ASCVD, 25.1% (515/2194) utilized some form of HIT (Table 2). Adults without a history of ASCVD utilized at least one form of HIT at a significantly higher proportion, that is, 51.0% (123,966/253,923). There were significant differences observed between adults with and without a history of ASCVD in 4 of the 5 individual indicators of HIT use (looking up health information on the internet, filling a web-based prescription, scheduling a medical appointment on the internet, communicating with a health care provider through email), with adults without a history of ASCVD utilizing these services more than adults with a history of ASCVD (Table 2).

**Table 2:** Weighted percentages and standard errors of health information technology use by adults with and without history of atherosclerotic cardiovascular disease.

Health information technology use indicators	All^a^, % (SE)	Adults with ASCVD^b^ (n=2194), % (SE)	Adults without ASCVD (n=253,923), % (SE)	*P* value
Overall health information technology use^c^	50.76 (0.2)	25.07 (1.1)	50.97 (0.2)	<.001
Looked up health information on the internet	48.72 (0.2)	23.13 (1.1)	48.94 (0.2)	<.001
Filled a web-based prescription	8.61 (0.1)	6.55 (0.6)	8.63 (0.1)	.004
Scheduled a medical appointment on the internet	9.53 (0.2)	4.70 (0.6)	9.57 (0.2)	<.001
Communicated with health care provider through email	10.38 (0.2)	5.39 (0.6)	10.42 (0.2)	<.001
Used online group chat to learn about health topics	3.50 (0.1)	3.35 (0.5)	3.50 (0.1)	.78

^a^Weighted percentage of all adults who used a form of health information technology.

^b^ASCVD: atherosclerotic cardiovascular disease.

^c^Use of at least one of the 5 indicators of health information technology.

**Figure 4:**
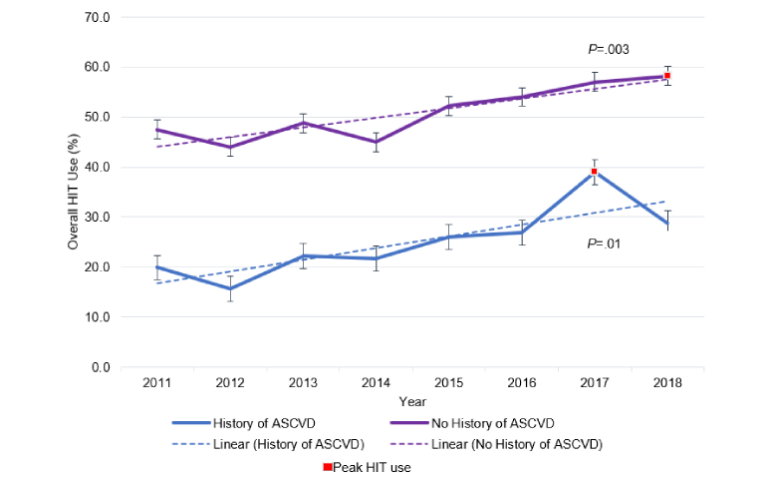
Trends in overall health information technology use among US adults with and without a history of atherosclerotic cardiovascular disease from 2011 to 2018. ASCVD: atherosclerotic cardiovascular disease; HIT: health information technology.

Figure 4 compares the prevalence of overall HIT use among US adults with and without a history of ASCVD from 2011 to 2018. HIT use among respondents with a history ASCVD demonstrated an upward trend over this period, peaking in 2017 at 39.0% (81/229). US adults without a history of ASCVD exhibited higher overall HIT use prevalence compared to adults with a history of ASCVD each year over the time period, peaking in 2018 at 58.2% (14,571/25,163). Table 3 displays the unadjusted prevalence ratios for HIT use during 2011 to 2018. An increased likelihood of HIT use over time was demonstrated among US adults with and without a history of ASCVD, with a general increase over the years and the most recent years of 2017 (with ASCVD: 1.96, without ASCVD: 1.20*)* and 2018 (with ASCVD: 1.45, without ASCVD: 1.23) exhibiting the highest likelihood of use.

**Table 3:** Prevalence ratios for health information technology use over time among adults with and without a history of atherosclerotic cardiovascular disease.

Survey year	Adults with ASCVD^a^ (n=2194), unadjusted PR^b^ (95% CI)	*P* value	Adults without ASCVD (n=253,923), unadjusted PR (95% CI)	*P* value
2011	1.00 (Ref)^c^	N/A^d^	1.00 (Ref)	N/A
2012	0.78 (0.52-1.18)	.24	0.93 (0.91-0.95)	<.001
2013	1.11 (0.80-1.56)	.51	1.03 (1.01-1.05)	.004
2014	1.09 (0.77-1.53)	.63	0.95 (0.93-0.97)	<.001
2015	1.31 (0.94-1.82)	.11	1.10 (1.08-1.12)	<.001
2016	1.35 (0.96-1.89)	.08	1.14 (1.11-1.17)	<.001
2017	1.96 (1.45-2.65)	<.001	1.20 (1.17-1.23)	<.001
2018	1.45 (1.04-2.01)	.03	1.23 (1.20-1.25)	<.001

^a^ASCVD: atherosclerotic cardiovascular disease.

^b^PR: prevalence ratio.

^c^Ref: reference.

^d^N/A: not applicable.

Persons with a history of ASCVD who used HIT were more likely to be females (25.9%) and younger in age, as the 18-29 age group had the highest prevalence at 74.7%. Likewise, persons with a history of ASCVD aged ≥70 years had the lowest prevalence of overall HIT use at 19.6%. Among the racial/ethnic groups, non-Hispanic White adults had the highest prevalence of overall HIT use (26.4%) and non-Hispanic multiple race and other race adults had the lowest at 17.1%. Persons with a history of ASCVD who exhibited higher prevalence in overall HIT use were more likely to have a higher income being at least >200% above poverty level (27.4%), be employed (33.1%), be married (29.8%), and have a bachelor’s degree or greater (44.2%). Significant differences in the prevalence of overall HIT use among adults with a history of ASCVD were found in most of the social determinants evaluated and are detailed in Table 4.

**Table 4:** Sociodemographic characteristics of the adults with a history of atherosclerotic cardiovascular disease who used health information technology (n=2194).^a^

Sociodemographic characteristics			Used HIT^b^, % (95% CI)		Unadjusted prevalence ratio (95% CI)		*P* value		Adjusted prevalence ratio^c^ (95% CI)		*P* value
**Gender**											
	Male	24.3 (21.5-27.0)		1.0 (Ref)^d^		N/A^e^		1.0 (Ref)		N/A	
	Female	25.9 (22.5-29.2)		0.9 (0.7-1.0)		.08		1.1 (0.9-1.3)		.40	
**Age (years)**											
	70+	19.6 (16.9-22.4)		1.0 (Ref)		N/A		1.0 (Ref)		N/A	
	60-69	29.2 (25.2-33.3)		*1.6 (1.3-1.9)*		<.001		*1.5 (1.3-1.8)*		<.001	
	50-59	33.3 (26.7-40.0)		*1.7 (1.3-2.2)*		<.001		*1.7 (1.3-2.2)*		<.001	
	40-49	42.2 (29.3-55.0)		*2.2 (1.6-3.1)*		<.001		*2.2 (1.6-3.1)*		<.001	
	30-39	46.1 (27.4-64.9)		*2.9 (1.9-4.6)*		<.001		*2.4 (1.6-3.7)*		<.001	
	18-29	74.7 (15.8-133.5)		*2.8 (1.1-7.1)*		.03		*3.9 (1.7-8.6)*		.001	
**Race/ethnicity**											
	Non-Hispanic White	26.4 (23.7-29.0)		1.0 (Ref)		N/A		1.0 (Ref)		N/A	
	Hispanic	23.4 (16.5-30.5)		*0.7 (0.5-1.0)*		.03		0.9 (0.7-1.2)		.43	
	Non-Hispanic Black	20.5 (15.9-25.1)		*0.7 (0.6-0.9)*		.01		*0.8 (0.6-1.0)*		.04	
	Non-Hispanic Asian	17.8 (9.4-26.1)		0.7 (0.4-1.3)		.31		0.7 (0.4-1.1)		.10	
	Non-Hispanic multiple races and other races	17.1 (1.7-32.6)		0.6 (0.2-1.5)		.24		0.6 (0.3-1.6)		.35	
**Poverty income ratio**											
	Below poverty level	20.7 (16.1-25.3)		1.0 (Ref)		N/A		1.0 (Ref)		N/A	
	Between 100% and 200% of poverty level	22.4 (18.4-26.5)		1.0 (0.8-1.4)		.89		1.1 (0.8-1.4		.56	
	>200% above poverty level	27.4 (24.4-30.5)		*1.8 (1.4-2.3)*		<.001		*1.3 (1.0-1.7)*		.03	
**Employment status**											
	Not employed	23.6 (21.3-25.9)		1.0 (Ref)		N/A		1.0 (Ref)		N/A	
	Employed	33.1 (27.6-38.6)		*2.5 (2.1-3.0)*		<.001		*1.4 (1.2-1.7)*		<.001	
**Marital status**											
	Not married	21.4 (18.8-24.1)		1.0 (Ref)		N/A		1.0 (Ref)		N/A	
	Married	29.8 (26.4-33.3)		*1.6 (1.4-1.9)*		<.001		*1.4 (1.2-1.6)*		<.001	
**Health status**											
	Better	24.3 (20.6-28.0)		1.0 (Ref)		N/A		1.0 (Ref)		N/A	
	Worse	26.3 (22.1-30.5)		0.8 (0.6-1.0)		.07		1.1 (0.9-1.3)		.49	
	About the same	24.6 (21.6-27.7)		0.8 (0.7-1.0)		.07		1.0 (0.8-1.2)		.90	
**Education level**											
	≤High school	12.0 (9.8-14.2)		1.0 (Ref)		N/A		1.0 (Ref)		N/A	
	Some college	34.9 (30.7-39.2)		*3.3 (2.6-4.0)*		<.001		*2.9 (2.3-3.6)*		<.001	
	≥Bachelor's degree	44.2 (38.5-50.0)		*4.4 (3.5-5.4)*		<.001		*3.7 (2.9-4.6)*		<.001	
**Insurance coverage**											
	Not covered	24.9 (22.7-27.1)		1.0 (Ref)		N/A		1.0 (Ref)		N/A	
	Covered	26.9 (14.8-36.0)		*1.6 (1.0-2.4)*		.04		1.1 (0.7-1.7)		.72	

^a^Italicized values are significant at *P*<.05.

^b^HIT: health information technology. HIT use is a composite variable coded using consecutive “OR” statements for the 5 indicators of HIT use measured in the National Health Interview Survey.

^c^Prevalence ratio adjusted for other sociodemographic characteristics.

^d^Ref: Reference.

^e^N/A: not applicable.

## Discussion

In this nationally representative sample of US adults with and without ASCVD, adoption of HIT among adults with a history of ASCVD was lower than that of HIT among adults without a history of ASCVD. HIT use prevalence was also lower among adults with sociodemographic characteristics that indicate vulnerability, such as unemployment and lower levels of education. Previous studies have found that older adults tend to be less likely to use the internet or search for health information online than younger adults [8,20]. Similar trends in HIT use in the older adult population were found in this analysis as the age groups of 60-69 years and 70+ years exhibited the lowest prevalence of HIT use among all age groups. There are several potential barriers to HIT use in the older adult population, such as limited health literacy, poor usability and accessibility of HIT, and impediments to effective use of HIT due to complications from chronic diseases such as vision impairment [21-23]. Care management and health promotion in older adults have been shown to be enhanced by HIT; thus, designing strategies that address these barriers could further enhance its effectiveness [24]. Younger family members and caregivers could be engaged to assist in HIT use.

Vulnerable populations such as those who earn lower incomes and are unemployed are more likely to suffer the consequences of the digital divide. People who are employed and have higher incomes are more likely to communicate with health care providers via text, phone apps, or social media [16]. This finding was corroborated in our analysis as the respondents that were >200% above the poverty level (poverty income ratio>2) and employed had a higher prevalence of HIT use compared to respondents that were below the poverty level and unemployed. Affordability of HIT is thus of major importance to increase access to potentially beneficial interventions that are currently barred by financial constraints. Education level has been cited as a determinant of HIT use in previous literature, with individuals with a college education being more likely to engage in eHealth behaviors compared to individuals without a college education [25]. Lower educational attainment has also been associated with lower health literacy, and this has resulted in individuals seeking self-management support either in person or by phone rather than through the internet [26]. Our analysis was consistent with these findings as respondents with lower educational attainment (a high school education or less) had the lowest prevalence of HIT use. HIT strategies should address gaps linked to health literacy as patients with sufficient health literacy are more likely to have access to the internet at home, search the web, access health information via the internet, email via the internet, and communicate with health care providers than patients with marginal to low health literacy [27].

Evaluating HIT use by race/ethnicity has been a major area of interest in terms of bridging the gap of the digital divide. Previous studies have reported that in both outpatient and inpatient environments, Black and Hispanic people are less likely to adopt and use patient portals [28,29]. A nationally representative study examining the demographics of users of health-related information obtained via the internet found that users were more likely to be White or Asian people [16]. Our analysis of the US adult population with ASCVD also found that there was a statistically significant lower prevalence of HIT use among Non-Hispanic Black people (prevalence ratio of 0.8, *P*=.04) but not Hispanic people (prevalence ratio of 0.9, *P*=.43) compared to White people (reference with prevalence ratio of 1.0) when adjusted for other sociodemographic characteristics. However, HIT use in this analysis did not include mobile devices. In recent years, mobile phones have emerged as a method to bridge the digital divide as they can provide access to internet services. Blacks are more likely to use a mobile phone to search for health information via the internet, and these devices could be used for targeted interventions [8].

An analysis using data from the 2012 and 2014 Health Information National Trends Survey measured preferences and use of HIT among US adults with and without 3 chronic disease conditions (CVD, diabetes, and hypertension) [7]. After adjusting for sociodemographic characteristics, the analysis found no significant association between these cardiovascular comorbidities and HIT use, suggesting that sociodemographic factors may have a greater influence on the adoption of HIT than the chronic diseases themselves [7]. Our analysis among US adults with a history of ASCVD that adjusted for sociodemographic variables did find significant differences in the prevalence of overall HIT use. The difference in the overall use of HIT further highlights that our fundamental approach to launching these technologies should account for the socioeconomic challenges they may face. This study demonstrates the influence that sociodemographic characteristics have on HIT adoption. It is important for clinical and public health professionals to incorporate the social determinants that impact patients’ health in the design of novel HIT to facilitate effective use. Emerging technologies for patients with ASCVD, such as mobile health interventions, telemedicine, and artificial intelligence have the potential to help manage CVD risk factors, reduce rehospitalizations from cardiac causes, and lower overall health care costs [5,30]. Addressing sociodemographic barriers to HIT use in a population with ASCVD can help ensure that these digital interventions meet the needs of these patients at scale.

This study has the following strengths. The NHIS is a large nationally representative survey, and this analysis pooled 8 years of data to increase the statistical power of the models for more accurate comparisons among the study population. However, health conditions such as coronary heart disease and stroke were self-reported, which could result in an underestimation of HIT use among this population if some individuals misclassified their condition. The smaller sample sizes for adults within our selection criterion in the race/ethnicity group, Non-Hispanic multiple races and other races, and the age group of 18-29 years may result in our estimates being less precise for those groups. CVD risk factors such as diabetes mellitus, hyperlipidemia, smoking, and hypertension could also serve as additional indicators of ASCVD outcomes and be included in future models assessing HIT use among the ASCVD population.

This study has the following weaknesses. HIT use was defined as using a computer in the past 12 months to perform a health-related task, and this did not include other mobile devices. According to the analysis of the 2018 Health Information National Trends Survey by Shan et al [17], 92% of US adults with or at risk of CVD owned a cell phone and 81% owned a smartphone in 2019. Mobile health devices have the potential to consolidate these services for patient-clinician communication, and their absence in the assessment may have resulted in an underestimation of use.

In conclusion, overall HIT use was low (25%) among adults with self-reported ASCVD, which may represent a barrier to delivering care via emerging HIT. Adults with ASCVD who were older, less educated, unemployed, racial minorities, not married, and had lesser income showed a lower prevalence of overall HIT use. To scale HIT interventions such as telemedicine, targeted strategies are needed to address the sociodemographic barriers to HIT adoption.

## Abbreviations

ASCVD: atherosclerotic cardiovascular disease
HIT: health information technology
NCHS: National Center for Health Statistics
NHIS: National Health Interview Survey
